# Extracellular Vesicle Mediated Crosstalk Between the Gametes, Conceptus, and Female Reproductive Tract

**DOI:** 10.3389/fvets.2020.589117

**Published:** 2020-10-30

**Authors:** Ahmad Yar Qamar, Feriel Yasmine Mahiddine, Seonggyu Bang, Xun Fang, Sang Tae Shin, Min Jung Kim, Jongki Cho

**Affiliations:** ^1^College of Veterinary Medicine, Chungnam National University, Daejeon, South Korea; ^2^Department of Clinical Sciences, College of Veterinary and Animal Sciences, Jhang, Sub-Campus University of Veterinary and Animal Sciences, Lahore, Pakistan; ^3^Department of Theriogenology and Biotechnology, College of Veterinary Medicine, Seoul National University, Seoul, South Korea

**Keywords:** intracellular communications, biofluids, functional molecules, embryo, placenta

## Abstract

Extracellular vesicles (EVs) mediated intracellular communication plays an imperative role in the proper completion of different physiological events. Most of the bio-fluids are enriched with several subpopulations of EVs including exosomes and microvesicles (MVs), with the capacity of transferring different functional molecules (lipids, proteins, and nucleic acids) to target cells. Recipient cells upon receiving the signal molecules undergo different changes that positively affect the structural and functional integrity of the cells. This article was aimed to highlight the role of EVs secreted by gametes, the female reproductive tract, and the growing conceptus in the successful completion of different reproductive events related to gestation. EVs associated with the reproductive system are actively involved in the regulation of different physiological events including gamete maturation, fertilization, and embryo and fetal development. In the reproductive system, EVs mediated intracellular communication is not unidirectional but is rather regulated through crosstalk between the reproductive tract and the growing conceptus. These vesicles are secreted from the ovary, oviductal epithelium, endometrium, developing embryo, and the placenta. The cargo inside these vesicles exerts pleiotropic effects on both maternal and embryonic environments. A better understanding of the EVs-mediated crosstalk will be helpful in the development of useful tools serving both the diagnostic as well as therapeutic needs related to female fertility.

## Introduction

Intercellular communication is considered imperative for the regulation and accomplishment of different physiological events including cellular proliferation and differentiation, gametogenesis, fertilization, and embryonic development (1). The success of pregnancy greatly depends on gametogenesis, fertilization, and an adequate uterine environment for embryonic development (2). These highly complex processes greatly rely on the crosstalk between the gametes and the different segments of the reproductive tract. The cells comprising a multicellular organism adopt different strategies to communicate with each other, including direct contact *via* membrane-bound signaling molecules and paracrine signaling *via* chemokines, cytokines, and hormones. Recently, an alternative mechanism has been described for intercellular communication involving the active participation of extracellular vesicles (EVs) that regulate diverse signaling pathways in targeting the cells (3, 4).

EVs are lipoproteinaceous membrane-enclosed nanometer-sized structures (5) released by almost all types of cells through different means of biogenesis. Moreover, these semi-spherical vesicles contain water-soluble components enclosed by a lipid bilayer, comprised mainly of disaturated lipids including gangliosides and sphingomyelin (6). EVs serve as a vehicle for the transportation of different proteins, lipids, RNAs (lncRNA, mRNA, small non-coding RNA, rRNA, and miRNA), and DNAs (dsDNA, ssDNA, and mtDNA) either locally or remotely (7, 8). The concentration of EVs and their cargo composition largely depend on physiological and environmental conditions (9). The contents of these vesicles can affect different physiological and pathological conditions through epigenetic and phenotypic modifications of both donor and target cells (10, 11). Furthermore, they actively participate in different biological activities such as immunomodulation, transportation, and propagation of infectious cargo including prions and retroviruses (12, 13), reproduction, candidate biomarkers of health and disease, and potential targets for therapeutics (14).

EVs are considered miniature versions of cells as they maintain the same orientation inside and outside the donor cells (15). Previously, EVs were classified based on origin, shape, size, and structural components as exosomes (40–100 nm), a product of multivesicular endosome (MVE) fusion (16, 17); microvesicles (MVs) or ectosomes (100–1,000 nm) that bud from the cell surface (18); oncosomes (1–10 μm) that protrude from the surface of cancer cells (19, 20); and apoptotic blebs (1–2 μm) released by cells undergoing apoptosis (21–23). However, the classification of EVs subtypes has been changed due to contradictory definitions and inaccurate expectations of unique biogenesis. According to the recent classification, EVs subtypes include small EVs, medium/large EVs, low-/middle-/high-density EVs, Annexin A5-stained EVs, CD63+/CD81+ EVs, podocyte EVs, hypoxic EVs, and large oncosomes (24). These vesicles have been isolated from physiological fluids including amniotic fluid (25, 26), ascites fluid (27), bile (28), blood (29), breast milk (30), cell cultures (31), cerebrospinal fluid (32, 33), plasma (34), saliva (35, 36), semen (37–39), urine (40, 41), and pathological effusions of the tumor (27). EVs secreted by donor cells can travel through the body fluids to deliver their contents to the target cells without degradation.

Recently, EVs such as exosomes and MVs have been isolated from the different segments of the male and female reproductive systems. EVs derived from the reproductive system regulates different reproductive events such as sperm maturation (42, 43), sperm capacitation and acrosome reaction (44, 45), oocyte maturation (46), fertilization (1), recognition of pregnancy (47, 48), implantation of pregnancy (49, 50), maintenance of pregnancy, and parturition (51). Moreover, EVs have been reported to regulate different reproductive pathologies such as early pregnancy loss, endometriosis, erectile dysfunction, gestational diabetes mellitus, gestational vascular complications, hypertension, polycystic ovaries, and preeclampsia (52). These pathological conditions may compromise fertility and even result in termination of pregnancy. EVs associated with the different phenomenon of reproductive physiology and pathology can potentially severe as biomarkers and vectors for drug delivery. Recently several reports have elucidated the effectiveness of EVs as mediators of the different phenomenon of reproductive physiology. The objective of this review is to highlight the potential roles of EVs in maternal reproductive physiology and their implications on successful pregnancy.

### EVs and Sperm Maturation

Mammalian testicles produce fully differentiated sperm; however, they lack motility and fertility. Sperm become fully functional as they transit through the different segments of the male reproductive system (53). EVs secreted by the male reproductive tract including epididymosomes and prostasomes play a key role in the maturation process of sperm (40, (54)). Moreover, prostasomes contain chromogranin B responsible for the bactericidal activity and prevent immune cells to recognize sperm in the female reproductive tract (55). On the other side, after coitus sperm are exposed to the different segments of the female reproductive tract as they move from the vagina to the fertilization site after breeding. During their transit, the sperm plasma membrane experiences certain biochemical modifications causing remodeling of their surface molecules (56). These modifications occur possibly due to the crosstalk between the sperm and the female reproductive tract and are considered vital for functional maturity and fertility acquisition.

Recently, EVs have been isolated from the uterine (uterosomes) and oviductal (oviductosomes) fluids of murine (57, 58). These vesicles showed the expression of certain sperm essential proteins including sperm adhesion molecule 1 (SPAM1) and plasma membrane calcium pump (PMCA4). *In vitro* studies have confirmed the transfer of fertility regulating proteins from these EVs to sperm (57, 58). Moreover, recent studies have demonstrated the potential of EVs in preserving the structural and functional integrity of sperm against the damages induced during the freezing procedures (59, 60).

Uterosomes are EVs isolated from the uterine fluids (UFs) that are classified as both exosomes and MVs (40, 57). The cargo contained in uterosomes mainly includes nucleic acids such as mRNAs and miRNAs (61) and proteins such as SPAM1 and PMCA4a (57). Estrogen (E_2_) hormone is believed to be involved in regulating the expression of these macromolecules (40, 62). It was hypothesized that uterosomes are responsible for sperm capacitation, membrane stabilization (63), and final sperm maturation via miRNA transfer (64) (Table 1). Moreover, the presence of SPAM1 protein suggests the possible role of uterosomes in the inhibition of a premature acrosomal reaction during the uterine transit of the sperm (64).

**Table 1:** Production and physiological roles of extracellular vesicles (EVs) in female reproductive tract.

**Site/origin**	**Source**	**Specie**	**Type of EVs**	**Function**	**References**
Ovary	Cumulus, granulosa, thecal cells	Bovine and equine,	Exosomes and MVs	• Cellular proliferation and inflammatory response • Follicle growth, oocyte maturation and developmental competence	(65–67)
Oviduct	Oocytes and ZP	Mouse	Exosomes	• Transfer of CD9 and CD81 to the sperm plasma member helpful in oocyte-sperm fusion • Blocks polyspermy	(68, 69)
	Oviductal epithelial cells	Bovine	Exosomes and MVs	• Sperm capacitation and reduce polyspermy • Increase sperm motility and viability	(70, 71)
		Porcine	Exosomes and MVs	• Gamete maturation and fertilization	(72)
Uterus	Endometrial epithelial cells	Bovine, human, murine, and ovine	Exosomes and MVs	• Sperm membrane stabilization, maturation, and capacitation	(64)
				• Mediate trophoblastic cells to produce exosomes • Regulate the gene expression of both trophoblastic and endometrial cells • Implantation of embryo	(50, 73–76)
Conceptus	Embryonic trophectoderm	Ovine	Exosomes and MVs	• Interferon release that help in maintaining pregnancy	(47)
	Trophoblastic cells	Human	Exosomes and MVs	• Prevent immune rejection of conceptus due to Fas ligand	(77, 78)
	Amnion epithelial cell	Human	Exosomes and MVs	• Control inflammation of fetus • Inhibit immune activity *via* HSP72 production	(25, 26)
				• Initiation of parturition	(79)

*HSP72, heat shock protein 72; MVs, microvesicles; ZP, zona pellucida (80)*.

Based on the shape, size, and expression of surface markers, oviductosomes are characterized as both exosomes and MVs (81). The cargo packed inside the oviductosomes includes proteins αV integrin (cell surface receptor), CD9 tetraspanin (adhesion molecule), heat shock protein A8 (HSPA8, & PMCA4), lactadherin (P-aminosalicylic acid, PAS6/7; or milk fat globule-epidermal growth factor-8, MFGE8), oviductal specific glycoprotein (OVGP), lipids, SPAM1, RNAs, and miRNAs (80). Oviductosomes-derived proteins are involved in different physiological events associated with sperm, such as SPAM1 involved in acrosome reaction (64); OVGP, increases sperm viability and motility (Table 1) (70), reduces the incidence of polyspermy through zona hardening (71), induces the phosphorylation of sperm-associated proteins during capacitation (82, 83), and modulates fertilization (84); HSPA8, affects fertilization and early embryo development (85); HSP90, interacts with the zona pellucida (ZP) of oocytes (86); PMCA4, associated with Ca^2+^ ion efflux ultimately affecting motility and fertility (87); and lactadherin, involved in ZP binding (88).

### EVs and Oocyte Maturation

Ovarian follicles maturation and oocytes growth are highly associated (89) and considered crucial for effective embryo production (90). These highly complex processes are regulated by an extensive intercellular communication within the microenvironment of the follicle along with the functional and structural transformation of different cell types constituting the follicle (91). The crosstalk between oocytes and the surrounding follicular cells (theca and granulosa) is important for oocyte development (92), occurring mainly through gap junctions' proteins. Moreover, the follicular fluid comprising of different ions, metabolites, nucleic acids, and proteins, serves as an additional means of communication between oocytes and follicular cells. Oocytes achieve developmental competence through different signal transductions and molecular interactions that are mediated through the follicular fluid (93). These molecular interactions involve two-way traffic of certain crucial factors. These factors affect both the folliculogenesis and initiation of different signaling pathways involving different molecules such as insulin, transforming growth factor-beta (TGFB) and wingless/Int (WNT) signaling members (94, 95), growth factors (growth differentiation factor, GDF9; bone morphogenetic protein 4, BMP4) (96), and hormones (97).

Recent studies demonstrated the EVs including exosomes and MVs (small EVs) derived from the follicular fluid (65) and oocytes (68, 98). These vesicles mediate intercellular communication associated with follicular development and oocyte quality (65, 66, 99). In bovine, a negative correlation was reported between the exosomal concentration and follicular size (67). Moreover, the exosomes found in the bovine follicular fluid have been shown to affect the transcript abundance in oocytes and adjacent granulosa cells (100). Supplementation of *in vitro* maturation (IVM) medium with follicular fluid has been reported to increase the expansion of cumulus cells (101), stimulate granulosa cells proliferation (102), and enhance the blastocyst development rates (103). Rodrigues and his co-workers reported similar findings (104). The exosomal fraction of follicular fluid was believed to be the main reason for these improvements.

EVs indirectly affect the competence of oocytes by improving the function of cumulus cells (104). An *in vitro* study demonstrated the uptake of EVs in cumulus cells and their associated transzonal projections (103). During the early stages of oocyte maturation, transzonal projections extend from the cytoplasm of cumulus cells and mediate RNA transfer between cumulus cells and the oocyte (105). In addition to RNA, some researchers also propose that cytokines present in the exosomes regulate the various physiological aspects including proliferation and differentiation of cells, survival or atresia of follicles, and maturation of oocytes (106, 107). Recent studies confirmed the exosome-mediated transfer of miRNA (66) and mRNA (108) to bovine granulosa cells.

Follicular fluid exosomes have been reported to possess cytoprotective effects against stress (104). Under stressful conditions, the cellular contents are modified and the cell experiences an increased secretion of EVs that enhance the defense system and prevent cell death (109, 110). During oxidative stress, the granulosa cells-derived EVs showed a higher proportion of antioxidants and other substances associated with cellular defense as compared to the exosomes secreted during normal conditions (Table 2) (111). Similarly, another study identified the protective nature of follicular fluid exosomes against oxidative stress (108). *In vitro* treatment of oocytes with follicular fluid-derived exosomes reduced the apoptosis of cumulus cells and damage to oocytes caused by heat shock (104).

**Table 2:** *In vitro* studies involving the use of extracellular vesicles (EVs) isolated from luminal fluids of different segments of female reproductive tract.

**Source**	**Type of EVs**	**Process**	**Main findings**	**References**
Follicular fluids	Exosomes	Effects of bovine follicular fluid derived exosomes on the function of cumulus-oocyte complex	• Oocyte maturation: enhancing oocyte's functions, and protecting against stress	(104)
Granulosal cells	Exosomes	Effect of oxidative stress on the cellular and exosome mediated defense mechanism of granulosal cells	• Oxidative stress leads to the activation of cascades involving cellular antioxidants released into extracellular environment via exosomes	(111)
Oviductal epithelial cells	Exosomes and MVs	Effect of MVs or miRNAs in maturation of canine oocytes	• Oviductal MVs may be involved in cellular trafficking during oocyte maturation	(112)
		Effect of supplementing IVF and IVC medium with porcine oviductal fluid isolated EVs	• Reduced polyspermy via zona hardening and improved developmental competence of embryos	(81)
		Effect of bovine oviductal derived EVs on bovine IVP	• Improved embryo quality and their cryotolerance	(113)
		Effect of oviductal derived EVs on the development and quality of bovine embryo	• Improved embryonic development and quality	(88, 114)
		Effect of oviductal derived EVs on embryo transfer efficiency of mice	• Improved embryo transfer efficiency due to reduced cellular apoptosis and better differentiation	(115)
Uterine fluids	Exosomes	Supplementation of bovine IVC medium	• Improved embryonic development, hatching, and quality. Improved gene expression	(116)
Conceptus	Exosomes and MVs	Analysis of *in vitro* embryo culture medium	• High expression of pluripotency related gene (Oct, Sox2, Klf4, c-Myc, and Nanog) Mediate cellular adhesion and migration	(117)
		Interaction between embryo and oviductal cells	• Embryo-derived exosomes modulate the oviductal cells through HSP10 and miRNA	(118)
		Coculture of parthenogentic and cloned embryos	• Improved cleavage, blastocyst development and quality of cloned embryos Improved the expression of *Oct4, Klf4*, and *Nanog*	(119)
		Isolation of EVs from IVC medium of bovine SCNT embryos	• Essential for development and quality of blastocysts	(120)
		Role of p38 MAPK associated with fetus-derived exosomes	• Influence parturition through inflammatory response, cell proliferation, apoptosis, and stress	(121, 122)
	Embryonic stem cells derived EVs	Effect of embryonic stem cells derived EVs on trophoblast	• Promote the migration and enhance the implantation	(123)

*EVs, extracellular vesicles; IVC, in vitro culture; IVF, in vitro fertilization; MAPK, mitogen-activated protein kinase; MVs, Microvesicles*.

**Figure 1:**
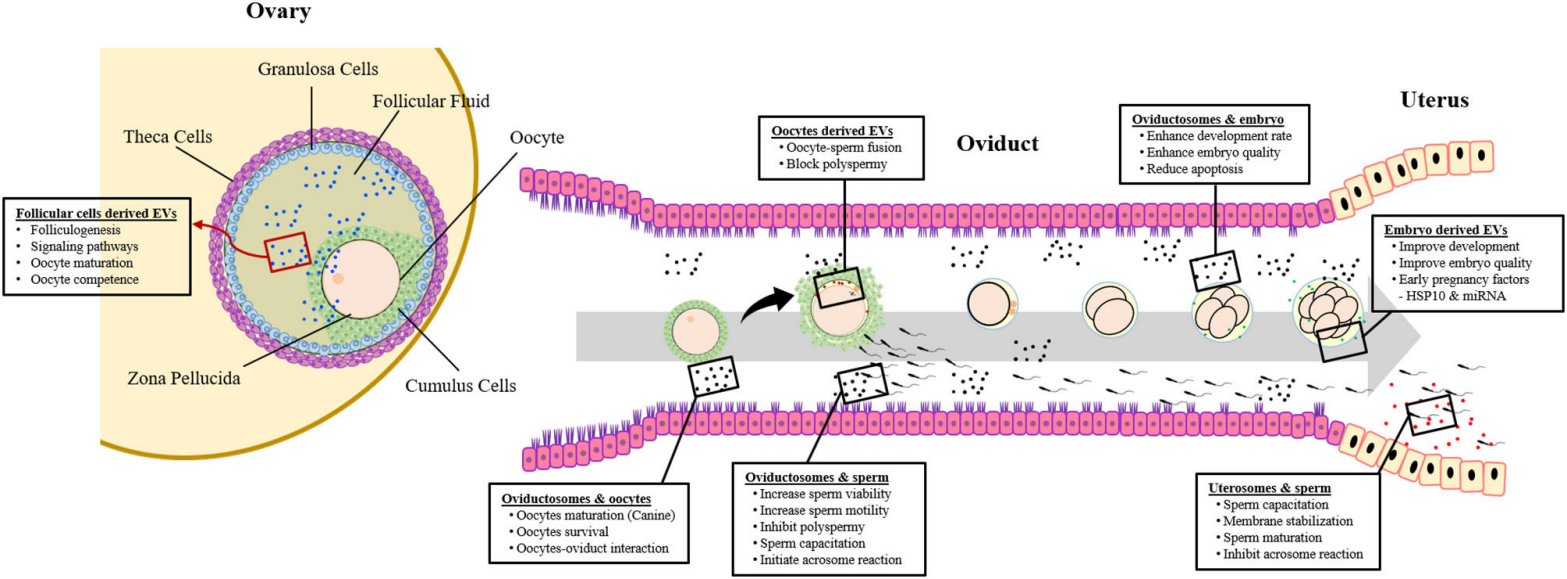
Extracellular vesicles (EVs) and crosstalk between gametes, embryo, and maternal reproductive system.

Oviductosomes are considered as key players involved in the regulation of the gametes-oviduct interaction (64, 88). In contrast to other mammalian species, the canine oocytes are ovulated in an immature form. For acquiring developmental competence, oocytes need both intra- and extra-follicular (oviduct) environments (124). Therefore, the role of oviductosomes in oocytes maturation and survival may be more pronounced in canines as shown in Figure 1. A recent study revealed that supplementation of IVM medium with oviduct-derived MVs was beneficial to the maturation of canine oocytes as involved in cellular communications (112). It was found that MVs were uptake by ooplasm after 72 h of maturation, demonstrating their role in improved maturation.

### EVs and Fertilization

The fertilization process involves the fusion of haploid gametes (male and female) within the microenvironment of the oviduct to produce a genetically distinct individual. Fertilization consists of different events occurring in a precise order, such as the expansion of cumulus cells to facilitate sperm passage, attachment of sperm with ZP (125), and finally penetrating ZP. The acrosomal reaction is initiated as capacitated sperm binds with the ZP resulting in the biochemical modification of the sperm plasma membrane. All these changes enable the sperm to recognize the ZP and allows the entry of sperm into the perivitelline space (126–129). Within the perivitelline space, the sperm fuses with the oocyte's membrane resulting in the depolarization of ZP. The depolarized ZP triggers the cortical granules to release their contents such as plasminogen, acid phosphatase, peroxidases, etc., which helps to avoid polyspermy.

Reports indicate that oocytes secrete a limited number of EVs that interact with the other cells present in the close vicinity of the oocyte (55). During oocyte-sperm fusion, oocyte-derived EVs are secreted into the perivitelline space (68) and ultimately transferred to the sperm plasma membrane. Transfer of oocytes-derived EVs to the sperm plasma membrane is considered vital for oocyte-sperm fusion (Table 1). Tetraspanins including CD9 and CD81 have been reported to mediate the oocyte-sperm fusion process. Before fusion, CD9 primarily produced by the oocyte is mainly localized in the oocyte's plasma membrane especially in the region of the microvilli, whereas CD81 produced by the cumulus cells is localized in the inner region of ZP (130). When sperm penetrates the perivitelline space both CD9 and CD81 are transferred to the sperm plasma membrane *via* EVs (130, 131). The suggested role of CD9 in the regulation of gametes fusion was well-supported by the fact that CD9 deficient oocytes have structurally altered microvilli and cannot fuse with the sperm (132). CD81 participates in fertilization, especially in fusion-related events like acrosome reaction (133). It is also believed that CD81 may facilitate the transfer of CD9 from the oocytes to the sperm plasma membrane (130).

Masaru Okabe's group identified Izumo 1 (named after a Japanese shrine), a member of the tetraspanin family, to be associated with oocyte-sperm fusion (134). Izumo 1 is localized on the surface of sperm that had undergone the acrosomal reaction. A deficiency of Izumo protein results in the failure of sperm to fuse with the oocyte. In an attempt to understand the role of Izumo in oocyte-sperm fusion, researchers identified the binding partner of Izumo on the surface of an oocyte named Juno (69). In the same study, it was found that the interaction between Izumo and Juno receptors is essential for mammalian fertilization, as oocytes lacking Juno are unable to fuse with the normal acrosome reacted sperm. Moreover, Juno receptors are involved in the prevention of polyspermy. Once fertilization occurs, the receptivity of fertilized oocyte shuts down for the additional sperm. Juno receptors are highly expressed on the surface of oocytes before fertilization. The fusion of gametes is followed by the shedding of these receptors from the oocyte's membrane and redistribution within the field of vesicles localized in the perivitelline space. It is believed that Juno conjugated vesicles bind and neutralize the incoming acrosome reacted sperm (69).

A recent study demonstrated that the porcine oviductosomes showed the expression of oviduct specific glycoprotein (OVGP1), ribosomal protein S5 (RPS5), myosin heavy chain 9 (MYH9), and valosin containing proteins (VCP) (81). OVGP1 in association with MYH9 can bind with the gametes (72), indicating their possible role in gamete maturation and fertilization. Whereas, RPS5 and VCP proteins are involved in protein synthesis (135, 136). The same study revealed that the supplementation of the *in vitro* fertilization (IVF) medium with porcine oviductosomes reduced the incidence of polyspermy by the hardening of ZP. Similar findings were observed when the IVF medium was supplemented with porcine oviductal fluids (137, 138) or conditioned medium isolated from oviductal cells culture (139). The glycoprotein structure of ZP was modified by either OVGP1, lactoferrin, and osteopontin binding (140) or OVGP1 and MYH9 proteins derived from EVs coupled together and bind with ZP to exert their effect (81).

### EVs and Preimplantation Embryonic Development

The physiological conditions during early embryonic development are considered crucial as their effects are translated in the subsequent growth. A successful pregnancy requires synchronized communication between the embryo and the female reproductive system. Any disturbance in this communication can either result in defective growth or lead to pregnancy termination (141). Recent studies have confirmed the active involvement of EVs in embryo-maternal cross talks. EVs identified in oviductal (44, 114) and uterine (50, 61) fluids, regulate the embryo-maternal interactions (64, 73). The composition of EVs contents varies according to the stage of pregnancy (74). Moreover, the preimplantation embryos are believed to produce EVs based on the high levels of EVs isolated from the embryo culture medium (119, 142). However, some research groups deny this fact, as they believe that EVs isolated from the culture medium may arise from the nutritional supplements used for embryo growth.

#### Oviduct-Derived EVs and Embryonic Development

Being the fertilization site, the oviduct represents the first environment to which embryos are exposed and also the site where embryo-maternal interactions start (143). Oviduct-derived EVs play an important role in mediating the embryo–maternal interactions during early embryonic development, leading to improved embryo quality and successful pregnancy (Figure 1). Almiñana et al. demonstrated the production of oviductal fluid EVs during both *in vivo* and *in vitro* conditions (88). Further experiments revealed an enhanced development rate and quality of embryos following supplementation of *in vitro* culture (IVC) conditions with *in vivo*-derived oviductosomes. It was concluded that *in vitro*-derived embryos can uptake the *in vivo* derived EVs from oviductal epithelial cells, resulting in improved competence. Similarly, improved development, quality, and cryo-tolerance of *in vitro* produced embryos were observed using EVs derived from bovine oviductal epithelial cells both *in vivo* and *in vitro* conditions (113, 114). Furthermore, the oviductal cells-derived EVs improved the efficiency of embryo transfer in mice, through reduced apoptosis and better differentiation of embryonic cells (115).

#### Uterus-Derived EVs and Embryonic Development

Following early embryonic development in the oviduct, the morula stage embryo enters the uterus on day 5 in bovine (144), and day 4–6 in ovine (73). Once in the uterus, the preimplantation embryo is dependent on the UFs secreted by the glandular cells lining the uterus (145). UFs contains amino acids, growth factors, lipids, proteins (146), and EVs containing bioactive substances required for embryonic development (50, 55, 147). EVs isolated from endometrial-epithelial cells and uterine mucus not only facilitate endometrium-embryo crosstalk but also help in the implantation of the embryo (50).

**Figure 2:**
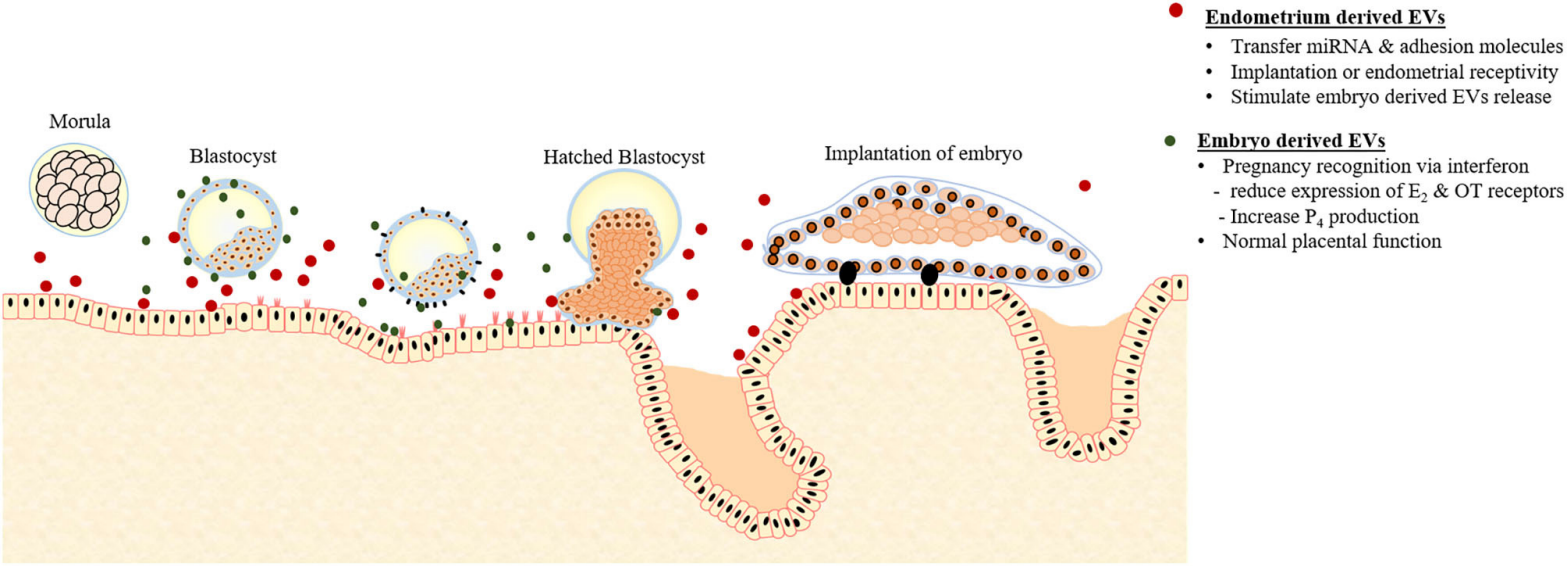
Extracellular vesicles (EVs) mediated crosstalk between early embryo and endometrium responsible for pregnancy recognition and implantation.

As the embryo arrives in the uterus intense embryo-maternal crosstalk starts, necessary for the maternal recognition of pregnancy. Burn and his coworkers demonstrated that during the early stage of pregnancy (15–17 days) in sheep, both trophectoderm and uterine epithelial cells actively communicate with each other via EVs (73). The endometrial epithelial cells of pregnant sheep produce exosomes containing endogenous jaagsiekte retroviruses (enJSRV) mRNA that causes the trophoblastic cells to release EVs containing interferons (47). As illustrated in Figure 2, interferons are cytokines having paracrine activity; causing reduced expression of estrogen (E_2_) and oxytocin receptors essential for the life of corpus luteum (CL) and progesterone (P_4_) production (148). P_4_ is a steroid hormone secreted by the CL required for pregnancy maintenance, elongation, and survival of conceptus (149). In addition to pregnancy supporting functions, P_4_ influences the secretions of EVs from the endometrial epithelial cells. In sheep, increased secretion of EVs from endometrial epithelial cells was observed during 10–14 days of the estrous cycle, the period of P_4_ dominance (150). Moreover, P_4_ up-regulates the miRNAs contents of EVs isolated from UFs that are supposed to modulate the BMP, phosphoinositide 3-kinase (PI3K), serine, or threonine kinase 1, and post-transcriptional silencing (150).

Furthermore, it was purposed that the EVs found in the UFs during early pregnancy might be involved in regulating the gene expression of trophoblastic cells and endometrial epithelial cells (75, 76). A recent study demonstrated that the supplementation of IVC medium with bovine UFs-derived EVs significantly improved the blastocyst development and hatching, and embryo quality in terms of cell number, and reduced apoptosis (116). Moreover, blastocysts treated with UFs-derived EV showed enhanced expression of interferon and acrogranin producing genes and reduced the expression of *BAX* and *HSP70* genes.

#### Embryo-Derived EVs and Embryonic Development

*In vitro* studies have revealed higher success rates of embryo production when culture frequency was kept higher throughout the culture period (151, 152), suggesting the possible existence of the embryo to embryo communication. Moreover, *in vitro* produced mammalian embryo can regulate its development independently until the hatching stage (153). It was believed that embryos develop their microenvironment through the release of different growth factors with autocrine and paracrine effects (142, 154, 155). Research evidence have demonstrated the isolation of EVs from the *in vitro* embryo culture medium on day 3 and 5 post culture (117, 156). Transcript analysis revealed high expression of pluripotency related genes including OCT4, SOX2, KLF4, C-MYC, and NANOG (Table 2). Moreover, the cargo was also comprised of a variety of miRNA species that mediate cellular activities such as adhesion and migration (Figure 2). The number of embryo-derived EVs increases with the developmental stage, whereas their size depends on the quality of the embryo (157).

A previous study suggested that the equine embryos at day 8 produce exosomes that interact and modulate the function of oviductal epithelial cells through the transfer of early pregnancy factor (HSP10) and miRNA *in vitro* (118). Similarly, the conceptus-derived EVs were isolated from the bovine UFs on days 15 and 17 of pregnancy (158). These EVs contained proteins that can modify the endometrial response and are unique to the existence of conceptus such as Aldo-keto reductase family 1, macrophage-capping protein (CAPG), member B1 protein (AKR1B1).

The cloned porcine embryos showed a significant increase in the development competence when co-cultured with parthenogenetic embryos (119). The co-cultured environment improved the cleavage and blastocysts formation rates, cell number, and higher expression of reprogramming related genes *OCT4, KLF4*, and *NANOG* than the embryos cultured alone (Table 2). Analysis of the culture medium of individual embryo showed the presence of CD9-positive vesicles. It was concluded that these vesicles are responsible for the transfer of mRNA cargos between embryos as these vesicles can cross the ZP and finally internalized by blastomeres. Another study about bovine revealed that embryo-derived EVs exert a positive effect on growth, survival, and pregnancy rates (120). In addition, EVs supplemented medium resulted in a significantly improved rate of blastocyst development and embryonic quality in terms of higher cell numbers.

### EVs, Embryo Implantation, and Fetal Development

#### EVs and Implantation

EVs derived from the endometrial epithelial cells play an active role in the transfer of signaling miRNAs and adhesion molecules either to blastocysts or to the adjacent endometrial cells affecting endometrial receptivity or implantation (Figure 2). The proteins contents of EVs derived from endometrium during the implantations stage contain a large number of adhesion molecules compared to the preimplantation stage EVs that contain proteins responsible for apoptosis (74). Moreover, treating endometrial epithelial cells with the EVs isolated from the UFs during the post-implantation period induced and up-regulated the expression of adhesion molecules such as vascular cell adhesion molecule 1 (VCAM) (74).

In a study, EVs isolated from the UFs of both cyclic and pregnant sheep reported containing miRNAs and proteins that are expressed by both trophectoderm of the conceptus and maternal endometrial epithelial cells (61). Similarly, another study identified that the endometrial epithelial cells are involved in the release of EVs in UFs. The cargo of these EVs includes specific miRNA populations such as has-miR-200c, has-miR-17, and has-miR-106a that are also found in the microenvironment of the embryo during implantation. It was hypothesized that these EVs may are involved in endometrial-embryo crosstalk during implantation (50). In mouse, the embryonic stem (ES) cells derived from the inner cellular mass (ICM) reported to secrete EVs containing fibronectin and laminin (123). These proteins interact with the integrin on the surface of the trophectoderm promoting the trophoblast migration. Furthermore, injecting ES cell-derived EVs in the blastocyst prior to injection in surrogate mother enhanced the implantation rate.

#### Placenta Derived EVs and Fetal Development

Following implantation, the placenta provides endocrine, nutritional, and oxygen support to the growing fetus. The placental cells including cytotrophoblasts, syncytiotrophoblasts, and mesenchymal stem cells reported to produce EVs that may also modify maternal physiology and fetal development (159). In sow, endometrial epithelial cells, maternal endothelial cells, chorioallantois, and trophectoderm secrete EVs on day 20th of pregnancy (160). Trophoblasts derived EVs carry molecules involved in the normal placental function. These EVs contain miRNAs and several important proteins that can modulate the angiogenesis within the trophectoderm. Moreover, the trophectoderm derived EVs penetrate and stimulate the proliferation of maternal endothelial cells. These molecules include fibronectin, which triggers pro-inflammatory events in early pregnancy (161); syncitin, associated with membrane fusion in syncytiotrophoblasts (162); Wnt/βcatenin-related molecules, involved in cell fusion (163); galectin-3, the interaction between placenta and endometrium (164); HLA-G5, immunosuppressive (162); prostaglandin E2, immunosuppressive; and 15d-PGJ2, a ligand to nuclear receptor PPAR_γ_ involved in parturition (165).

Placenta-derived EVs enter the maternal blood as early as the 6th week of gestation and are continuously secreted throughout the whole gestation (166). Taylor and his coworkers reported EVs in the maternal serum at 28–30 weeks of gestation (167). A recent *in vitro* study showed that the supplementation of IVC medium with pregnant sow serum inhibits apoptosis and promotes the cytoplasmic reorganization and differentiation of parthenogenetic embryos (168). Factors such as oxygen tension and glucose concentration regulate the release of EVs from the placenta. The concentration of placenta-derived EVs depends on the stage and physiological status of pregnancy. EVs concentration increases as pregnancy progress and is correlated with the mean blood flow through the uterine artery and weight of the placenta. During the first trimester of pregnancy, there is a 50-fold increase in the EVs concentration in maternal plasma (169). However, the plasma concentration of EVs and their associated bioactivity decreases in late pregnancy. EVs concentration was higher in the pregnancies that proceed normally till term than pregnancies that terminated preterm. Moreover, the concentration of placenta-derived EVs and their cargo composition is also associated with placental dysfunction. The EVs produced during the first trimester were more bioactive in promoting endothelial cell migration compared to the EVs produced during the second and third trimester (170). Therefore, fetal growth and placental function can be estimated by the quantification of EVs in maternal plasma.

**Figure 3:**
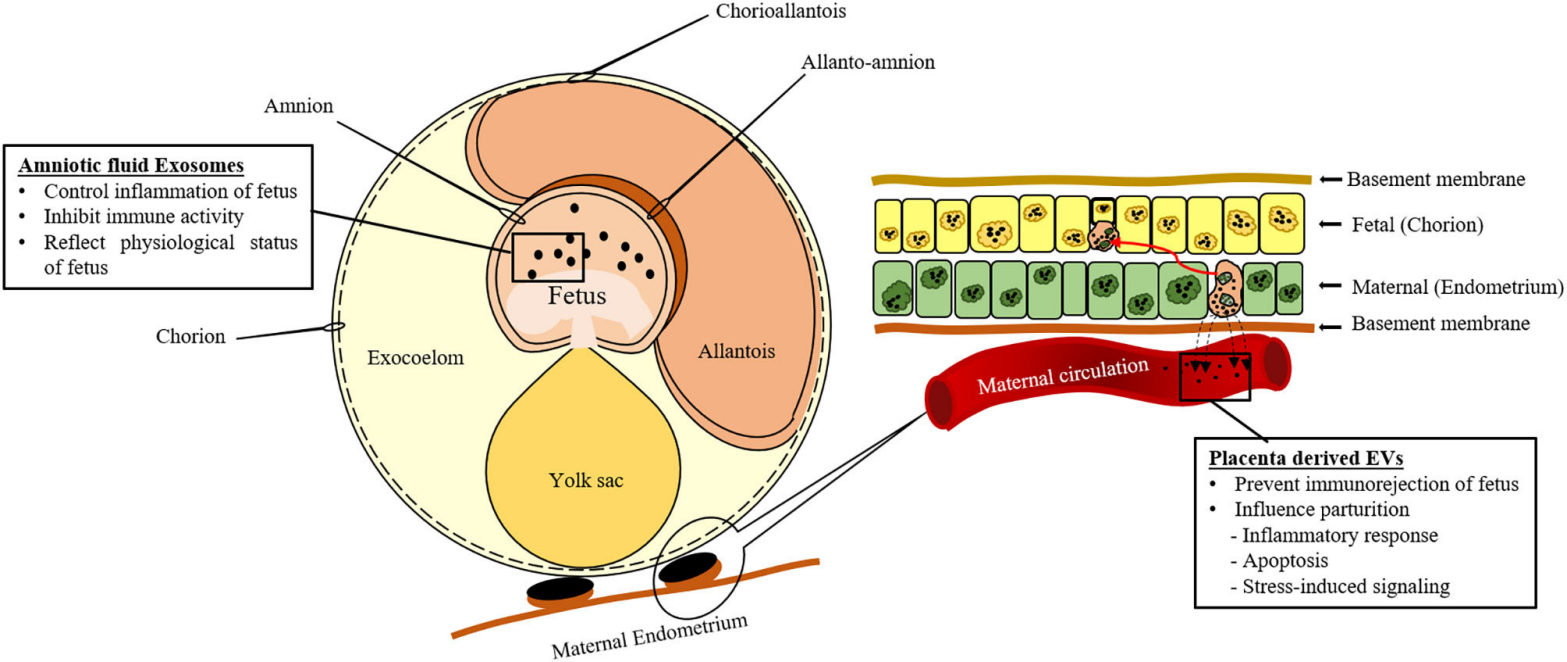
Schematic representation of extracellular vesicles (EVs) secreted by the conceptus during gestation.

Several studies showed excessive production of EVs during pregnancy with immunoregulatory properties for preventing the immuno-rejection of the fetus by the maternal immune system. During the first trimester, these vesicles contain Fas ligand (77, 78) that reacts with the maternal T lymphocytes to cause apoptosis and prevents anti-fetal immune response. Moreover, EVs also inhibit the expression of signal transduction molecules of T cells such as CD3 and JAK3 (167). The amniotic fluid is rich in EVs due to the secretion of fetal waste products and urine as shown in Figure 3 (25). It is believed that these EVs are involved in controlling the inflammation of different fetal compartments (Table 1). During the second trimester, EVs found in the amniotic fluid contain HSP72 that is a potent inhibitor of immune activity (Table 1) (26). In addition, amniotic fluid exosomes contents can reflect the physiological or pathological state of pregnancy around parturition (171). Amniotic fluid exosomes from different pathological conditions (spontaneous preterm birth, preterm premature rupture of membranes) and physiological conditions had different contents and exhibited different canonical pathways. These findings prove that amniotic fluid exosomes can be used as a diagnostic tool for preterm pathologies.

### EVs and Parturition

The mechanisms initiating parturitions are complex and involve both maternal and fetal sides. Leukocytes invasion, fetal endocrine factors, P_4_ withdrawal, and inflammation are the major events that trigger the switch of state from quiescent to active in gestational tissues, but little is known about the exact mechanisms involved in this transition. The hypothalamic-pituitary-adrenal axis is important to labor triggering factor and is already well-documented (172, 173). In mammals, except humans, parturition is initiated as serum P_4_ level drops down (174). In humans, the P_4_ level remains the same, but its function is blocked, a concept referred to as “Functional P_4_ withdrawal” (175). The withdrawal of P_4_ is followed by a cascade of endocrine changes, including E_2_ concentration increase, cortisol, prostaglandin, and oxytocin release, which ultimately lead to the cervical changes, uterine contractions, and membranes rupture (176). These events are also emphasized by the inflammatory reactions on the feto-maternal side (177) and EVs might play a role in these inflammatory processes.

EVs originate from both maternal and fetal sides and keep increasing throughout the pregnancy (178). These EVs carry pro-inflammatory factors that will subsequently influence the outcome of pregnancy by inducing pre-term or term parturition (169). On the maternal side, studies showed that parturition is associated with an increase in pro-inflammatory cytokines (179–181). One of the studies showed leukocyte invasion and increase mRNA expression of IL-1b, IL-6, and IL-8 in the cervix and myometrium near parturition (179). However, the cascade initiating this sterile inflammatory reaction was not investigated in these studies. Placenta-derived EVs might carry pro-inflammatory mediators that participate in the initiation of parturition, although research on the characterization of the different canonical pathways where these exosomes might be implicated are still ongoing (51).

Fetal cells exosomes from an amnion epithelial cell line can also induce an inflammatory reaction when delivered to myometrial and decidual cells (79). As fetal growth and maturation go by throughout the pregnancy, telomerase activity is reduced in fetal membranes, which leads to cellular aging and the expression of a senescence-associated secretory phenotype (SASP). It seems that oxidative stress-induced fetal cells would express senescence factors and thus inflammatory factors that would be conveyed through their exosomes to the maternal cells may initiate parturition. The cargo composition of the EVs derived from fetal cells exposed to oxidative stress were screened in another study (121). They demonstrated that p38 MAPK (mitogen-activated protein kinase) was found in high concentrations in the isolated EVs and the main factor influencing parturition. Moreover, p38 isoform is implicated in inflammatory responses, cell proliferation, apoptosis, and stress-induced signaling (122).

Regardless of their origin, late gestation exosomes release their cargo in specific sites, inducing inflammatory reactions in those sites only, subsequently avoiding systemic inflammation (178). The mechanisms behind the tropism exhibited by exosomes have not been elucidated yet, but Carboxyfluoroscein succinimidyl ester (CFSE)-labeled exosomes were localized in specific tissues: connective tissue of the maternal cervix, myometrium in the uterus, a labyrinth in the placenta and the epithelial layer in the fetal membranes. The same study showed that pre-term period exosomes, extracted at Day 18 of pregnancy, could influence the onset of parturition when injected on Day 15–16, regardless of the withdrawal of plasma P_4_ (178). This suggests that late gestation exosomes trigger the events that remodel and prepare the cervix and uterus for parturition (178).

The foregoing suggests that EVs are as important as endocrine factors in fetal-maternal communication especially in the initiation of parturition through their inflammatory effects. Pre-term exosomal cargo contains the highest level of inflammatory mediators when compared to the cargo from early pregnancy stage and the proteins contained there are implicated in biological functions that are essential to the late gestation period (leucocyte activation, neutrophils infiltration, chemotaxis, and cell movement) (178).

## Conclusions

This review highlights the biological roles of EVs in different events of female reproductive physiology associated with the pregnancy. There is growing evidence in the literature that the functional molecules carried by the EVs can modulate different reproductive events such as gametes maturation, fertilization, blockage of polyspermy, development, and implantation of the embryo, fetal development, and parturition. In addition, EVs can serve as excellent carrier for drug delivery due to the ability to transfer their contents to the target cells. Based on the physiological role, EVs supplementations can be used to overcome the deficiencies and enhance the outcomes of *in vitro* embryo production. Moreover, EVs concentration and their contents can reflect the physiological or pathological state of different reproductive events. It can be helpful in the estimation of pregnancy term, fetal growth, placental function, and diagnosis of different pathological conditions. However, the available data regarding the potential role of EVs in reproductive physiology and pathology is still limited and requires further investigations. As better understanding of EVs mediated communication can improve the diagnostics and therapeutics for fertility related issues, pregnancy-associated abnormalities, and pregnancy loss.

## Author Contributions

JC, MJK, and AYQ designed the manuscript, performed the literature review, and drafted the manuscript. JC and MJK supervised and critically reviewed the manuscript. AYQ, FYM, SB, SS, and XF participated in drafting, critically revising, and discussed the manuscript. All authors approved the manuscript for publication. All authors contributed to the article and approved the submitted version.

## Conflict of Interest

The authors declare that the research was conducted in the absence of any commercial or financial relationships that could be construed as a potential conflict of interest.
